# Coroner’s Inquests

**Published:** 1871-03

**Authors:** 


### Coroner's Inquests.

It is scarcely necessary to make a word of comment upon the communication of our worthy associate, Dr. Hauenstein, whose experience in this *post-mortem trade* is by no means serious as compared with the profession generally. Upon one of our patients, who died of well-known disease of the heart, two coroners held inquest. The first simply made inquiry as to the facts of his long sickness, and the number of physicians who had recognized the nature of the malady, and his liability to sudden death. The second called two physicians, who made *post-mortem* examination, and a jury to decide the cause of death. There was no suspicion of any crime, either by the dead man, his friends, or any body else. We were at the time unable to conceive the occasion for any inquest at all, much less for two inquests upon the same case of death from causes much better understood than is usual in the most common of fatal diseases. In another case we were summoned in haste to make examination for coroner. Judge, if you can, our astonishment, on entering the room, to find our own patient, who had long suffered from unmistakable cancer of the womb already dissected, and waiting our arrival, so as to give us opportunity of making "*post-mortem examination.*" Not very long since the cars run over a poor unlucky victim, and we were called to administer to his distresses the few remaining hours of his life. A few days later, the daily press announced the verdict of the coroner's jury, as follows: "Died from necessary surgical operation and loss of blood." As we are not very sensitive about such matters, we have hardly thought of it since, until Dr. Hauenstein's letter recalls our experience in this respect. Personally, we have no objection to coroners holding inquests on all who die; in many instances it might be well to determine whether death resulted from natural or unavoidable causes, or from neglect or imperfect care. Provided the county is willing to defray the expenses, there is no great objection; it might throw away its money in a worse way.

It seems quite inconsistant, however, that members of the medical profession should offer gratuitous medical care of the pauper population on the one hand and on the other render unnecessary and wholly useless service, with no other earthly motive except the compensation. The medical care of the poor is a legitimate service, which the county is legally and morally bound to requite. There can be no reason urged why physicians who render it faithfully and honestly should not be adequately paid. We believe no honorable tax-payer of the county would object to it, any more than to the salaries of the supervisors superintendents of the poor, and all others who devote their time to the county. It is no benevolence to offer it gratuitously: the county should not be willing to accept this service upon such terms from the men who offer it: they are not able to give their time to Erie County without compensation. It is disgraceful to both parties; alike injurious to the good standing and respectability of the one, and unbecoming the ability and fairness of the other. It is necessary in cases of suspected crime to make thorough investigation as to the causes of death.

Such services, when faithfully rendered, should also, be liberally paid; but it is in no way necessary, to examine by *post-mortem* section the causes of death, when there is no suspicion of crime, and when death has been long expected from the progress of common disease. That physicians should offer their services gratuitously where they are fully and fairly entitled to pay, and engage in an unnecessary, illegal and dishonest service to the same party, for the sole object of compensatisn, is indeed quite remarkable. Which is *most* dishonorable, we leave others to judge. It would be an interesting historical item, to know how many *post-mortem* examinations are ordered yearly by the Coroners of Erie County, and in how few of the number was any crime even so much as suspected

If any Coroner order *post-mortem* examination to determine cause of death, in cases of well understood and uniformly fatal disease, dying under the care of well-informed practitioners of medicine, it is malfeasance in office, and should be immediately presented to the courts for adjustment. Hereafter, we promise to the craft immediate complaint, so that we may have *ante-mortem* examination of their official conduct. Personally we entertain for them all the highest respect, and are under obligations for favors, which will be duly acknowledged under proper head.

